# Highly efficient PD-1-targeted CRISPR-Cas9 for tumor-infiltrating lymphocyte-based adoptive T cell therapy

**DOI:** 10.1016/j.omto.2022.01.004

**Published:** 2022-01-10

**Authors:** Christopher Aled Chamberlain, Eric Paul Bennett, Anders Handrup Kverneland, Inge Marie Svane, Marco Donia, Özcan Met

**Affiliations:** 1 National Center for Cancer Immune Therapy (CCIT-DK), Department of Oncology, Copenhagen University Hospital, Borgmester Ib Juuls Vej 25C, 2730 Herlev, Denmark; 2 Department of Odontology, Faculty of Health and Medical Sciences, University of Copenhagen, Nørre Allé 20, 2200 Copenhagen N, Denmark; 3 Department of Immunology and Microbiology, Faculty of Health and Medical Sciences, University of Copenhagen, Blegdamsvej 3B, 2200 Copenhagen N, Denmark; 4 Department for RNA & Gene Therapy, Novo Nordisk A/S, Novo Nordisk Park 1, 2760 Måløv, Denmark

**Keywords:** adoptive cell therapy, TIL therapy, tumor-infiltrating lymphocytes, CRISPR-Cas9, PD-1, immunotherapy, gene editing

## Abstract

Adoptive T cell therapy (ACT) with expanded tumor-infiltrating lymphocytes (TIL) can induce durable responses in cancer patients from multiple histologies, with response rates of up to 50%. Antibodies blocking the engagement of the inhibitory receptor programmed cell death protein 1 (PD-1) have been successful across a variety of cancer diagnoses. We hypothesized that these approaches could be combined by using CRISPR-Cas9 gene editing to knock out PD-1 in TILs from metastatic melanoma and head-and-neck, thyroid, and colorectal cancer. Non-viral, non-plasmid-based PD-1 knockout was carried out immediately prior to the traditional 14-day TIL-based ACT rapid-expansion protocol. A median 87.53% reduction in cell surface PD-1 expression was observed post-expansion and confirmed at the genomic level. No off-target editing was detected, and PD-1 knockout had no effect on final fold expansion. Edited cells exhibited few phenotypic differences and matched control functionality. Pre-clinical-scale results were confirmed at a clinical scale by generating a PD-1-deficient TIL product using the good manufacturing practice facilities, equipment, procedures, and starting material used for standard patient treatment. Our results demonstrate that simple, non-viral, non-plasmid-based CRISPR-Cas9 methods can be feasibly adopted into a TIL-based ACT protocol to produce treatment products deficient in molecules such as PD-1, without any evident negative effects.

## Graphical abstract

**Figure:**
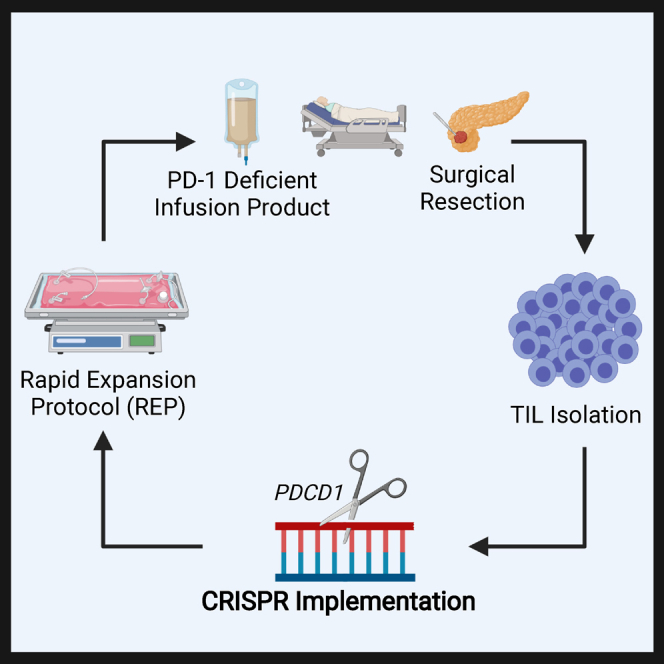

## Teaser

Chamberlain et al. demonstrate that CRISPR-Cas9 gene-editing techniques can be simply and effectively integrated into a TIL-based ACT workflow. Using an uncomplicated editing approach, they create a highly PD-1-deficient product with no detected off-target effects. This study can promote the application of these methods to improve TIL-based ACT.

## Introduction

Adoptive cell therapy (ACT) with ex-vivo expanded autologous tumor-infiltrating lymphocytes (TILs) has repeatedly mediated durable responses in patients with metastatic melanoma (MM),^1^ even after failure of prior immunotherapies.^2^, ^3^, ^4^ The success of TIL-based ACT in melanoma is yet to be replicated in additional cancer types, although recent reports have shown promise with both responses and durable complete responses demonstrated across a range of diagnoses.^5^, ^6^, ^7^, ^8^, ^9^, ^10^ Many efforts are being made to improve TIL-based ACT, primarily via selection and expansion of highly potent TILs^11^, ^12^, ^13^, ^14^; however, these methods can further complicate an already complex procedure and are often not suitable or successful for all patients. Therefore, there is a need for innovations that enhance TIL products for the majority of patients with minimal disruption to existing workflows.

Genetic manipulation of peripheral blood lymphocytes for ACT protocols has already been heavily investigated, with numerous ACT clinical trials employing gene-edited products either ongoing or already completed.^15^^,^^16^ Many of these studies include the removal of programmed cell death protein 1 (PD-1, *PDCD1*) from T cells, thereby preventing its interaction with programmed death ligand 1 (PD-L1) on antigen-presenting and tumor cells. Manipulation of this naturally homeostatic checkpoint interaction by tumors inhibits T cell-mediated tumor clearance, and therapies disrupting the PD-1/PD-L1 axis have been some of the most successful cancer treatments of the past decade.^17^^,^^18^ Despite these advances, many patients with tumor types commonly sensitive to manipulation of this axis either acquire resistance or do not respond these therapies.^19^ In addition, classical systemic treatment with anti-PD-1/anti-PD-L1 checkpoint inhibitors (CPIs) often induces unwanted immune-related adverse events by affecting T cells at non-tumor locations.^20^ Combining the demonstrated effectiveness of TIL-based ACT and PD-1/PD-L1 axis manipulation while potentially avoiding CPI-mediated side effects and resistance is therefore an appealing prospect.

Despite the discovery of powerful and easy-to-use gene-editing techniques such as CRISPR-Cas systems,^21^^,^^22^ and their widespread application in many ACT contexts, few studies have explored the possibility of applying these methods to TIL-based ACT. These pre-clinical studies have all targeted PD-1 and demonstrated encouraging editing efficiencies and potentially improved TIL functionality yet employed complex and costly workflows using transcription activator-like effector nucleases (TALENs) or zinc finger nucleases (ZFNs), which restrict the widespread application of these methods.^23^^,^^24^ In addition, these studies have focused on specific cancer types or achieved inconsistent efficiencies across cancer types.

In order to expand the implementation of gene editing in TIL-based ACT, we report here the generation of highly PD-1-deficient TILs for ACT across tumors of multiple histologies by using easily obtainable reagents and a simple, safe, clinically applicable, and CRISPR-Cas9-based workflow.

## Results

### PD-1-targeted CRISPR-Cas9 is compatible with a TIL-based ACT workflow

**Figure 1:**
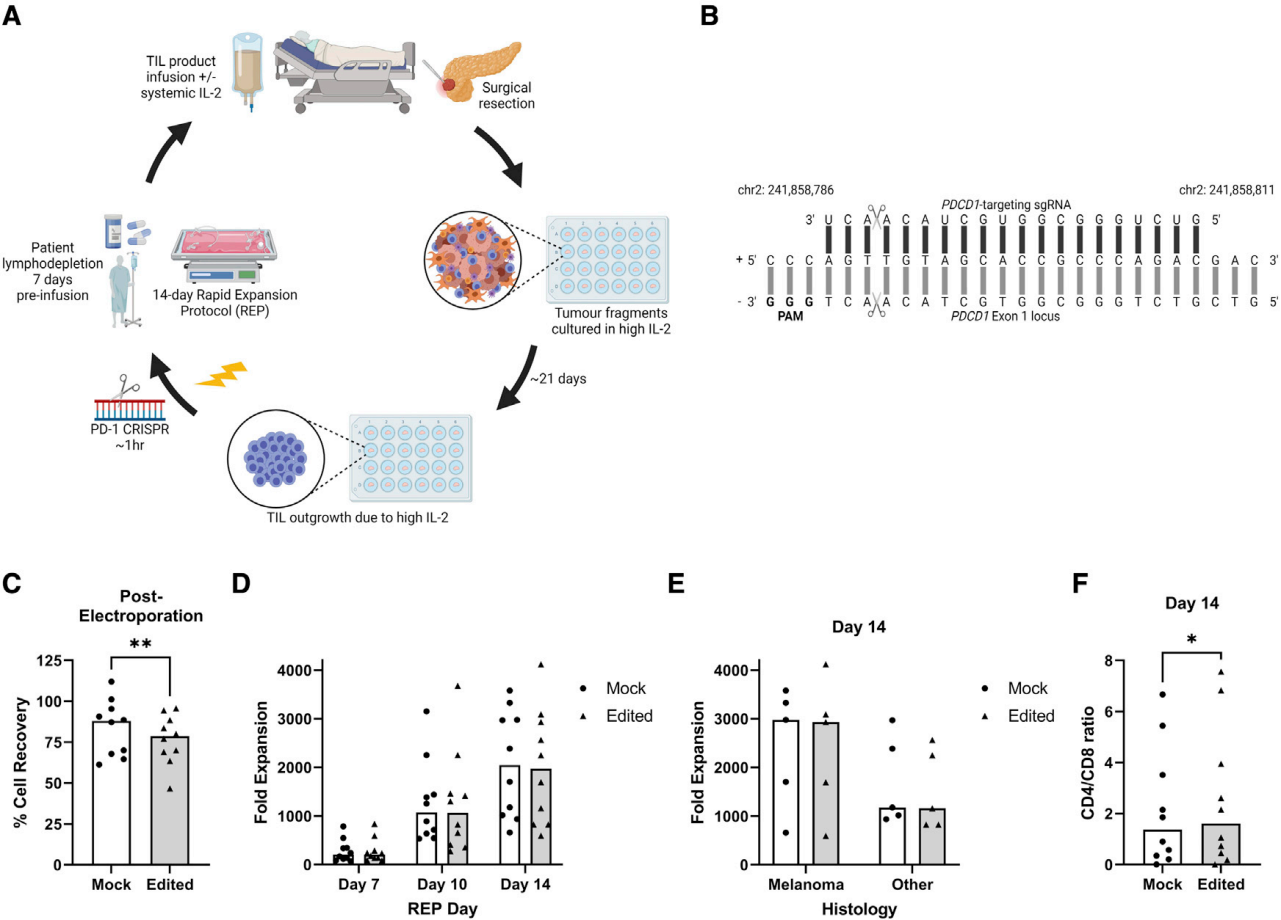
PD-1-targeting CRISPR-Cas9 is compatible with a TIL-based ACT workflow (A) Overview of the TIL-based ACT workflow from surgical resection to patient treatment, including the addition of PD-1 CRISPR-Cas9. (B) Base-pair level depiction of *PDCD1*-targeting gRNA binding to the target site (black bars). Expected cut site is marked with scissors, and the protospacer adjacent motif (PAM) for Cas9 binding is in boldface. Chromosomal location, strand direction, and sense are annotated. (C) Electroporation was used to deliver non- (mock) or *PDCD1*- (edited) targeting RNPs to pre-REP TILs. Cell recovery 1 h post-electroporation is shown. Statistical significance was calculated via paired t test (p = 0.0095). (D) Mock and edited pre-REP TILs were expanded with the 14-day REP protocol. TILs were counted, and fold expansion was calculated at days 7, 10, and 14. (E) Comparison of final fold expansions (day 14) for melanoma and non-melanoma REPs. (F) Calculated CD4/CD8 ratio in day 14 REP samples. Statistical significance calculated via paired t test (p = 0.0302). See also Figure S1. (B–F) Each point represents the average of two replicates per sample. Mock samples are shown as black dots on a white bar and edited samples as black triangles on a shaded bar. Bars signify median of 10 samples. (A–B) Created with BioRender.com.

Studies have confirmed the ability to integrate various genetic editing methods into the TIL-based ACT workflow^23^, ^24^, ^25^; however, this is yet to be demonstrated with CRISPR-Cas9-based editing methods. We therefore utilized a static pre-clinical-scale system modeling a clinical-scale patient-treatment workflow (Figure 1A) to test whether the application of CRISPR-Cas9 to TIL-based ACT was feasible and consistent across cancer types.^26^

Our optimal setup utilized the BTX ECM 830 Square Wave Electroporation System, approved for good manufacturing practice (GMP) use at our institute, to deliver CRISPR-Cas9 ribonucleoprotein complexes (RNPs) to TILs ∼1h prior to beginning the rapid expansion protocol (REP). Five MM and five non-MM (microsatellite stable [MSS]-colorectal, microsatellite instable [MSI]-colorectal, ovarian, head-and-neck, and thyroid cancer) samples were tested.

Delivery of *PDCD1*-targeting RNPs (Figure 1B) resulted in a minor (median 87.92% versus 78.57%, p = 0.0095) reduction in TIL recovery compared with mock controls 1h post-electroporation (Figure 1C). Despite this initial reduction in cell number, edited samples expanded as well as mock controls during the REP (median 2,044.5 versus 1,973-fold expansion, Figure 1D). On average, fold expansions in the non-MM group were considerably lower than in the MM group (median 1,168 versus 2,953.75); however, in both histology groups edited samples matched mock control sample fold expansions (median 2,932.5 versus 2,975 and median 1,160 versus 1,176; Figure 1E). A minor shift in the CD3+ cell CD4/CD8 ratio (p = 0.0302) in favor of CD4+ cells was detected in edited samples post-expansion (Figure 1F) and was confirmed to be mediated by an increase of CD4+ cells and a decrease of CD8+ cells (Figure S1).

### TIL-based ACT-compatible PD-1-CRISPR-Cas9 is stable and highly efficient

Having determined that *PDCD1*-targeting CRISPR-Cas9 can be included in a TIL-based ACT workflow, we next investigated the efficiency of the desired gene editing in the same pre-clinical-scale system. Previous pre-clinical studies targeting PD-1 in a TIL-based ACT context have reported average expression reductions of 76% using zinc finger nucleases (ZFNs) in melanoma^23^ and up to a 72% reduction using transcription activator-like effector endonucleases (TALENs) in breast cancer.^24^

**Figure 2:**
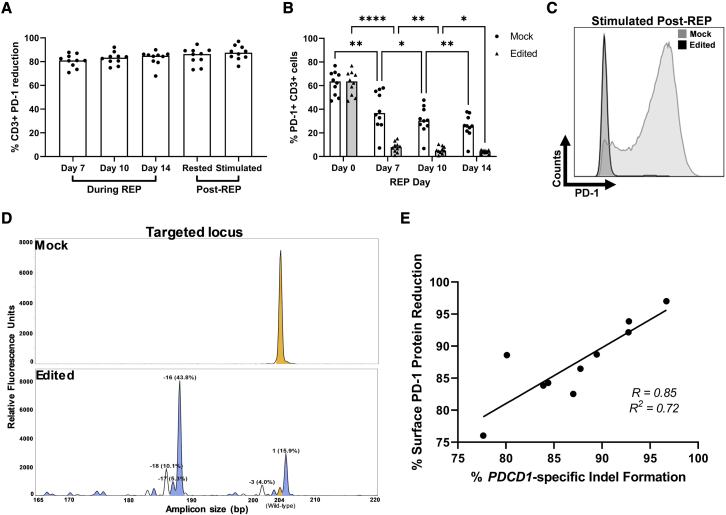
PD-1-targeting CRISPR-Cas9 for TIL-based ACT is highly efficient (A) CRISPR-Cas9-mediated PD-1 knockout throughout and post-REP measured via flow cytometry. Knockout calculated compared with Mock. See also Figure S2. (B) Surface expression of PD-1 on CD3+ TILs from mock and edited samples throughout the REP process, measured via flow cytometry. Statistical significance calculated by repeated-measures two-way ANOVA with Geisser-Greenhouse correction and Holm-Sidak multiple comparisons test (∗p ≤ 0.05, ∗∗p < 0.01, ∗∗∗p < 0.001, ∗∗∗∗p < 0.0001). See also Figure S2. (C) Representative histogram of PD-1 knockout measured by flow cytometry in post-REP stimulated sample. Mock sample shown as light gray and edited as black. (D) Representative REP day 14 IDAA plots for mock (top) and edited (bottom) samples. Yellow peaks denote unedited wild-type amplicons, blue peaks indicate frameshift indels, and white peaks indicate in-frame indels. (E) Correlation analysis of day 14 stimulated REP-TIL surface PD-1 knockout calculated via flow cytometry (compared with mock) and REP day 14 IDAA indel quantification. Correlation calculated via linear regression. Each point represents the average of two replicates for each of the 10 samples. (A and B) Each point represents the average of two replicates per sample. Bars signify median of 10 samples.

CD3+ cell surface PD-1 expression measured via flow cytometry was consistently reduced in edited samples throughout the REP, median 80.92%, 83.33%, and 84.96% reduction compared with mock on days 7, 10, and 14, respectively, and this reduction was maintained post-cryopreservation (median decrease 86.4%; Figures 2A, S2A, and S2B). Surface expression of PD-1 declined in both edited and mock control samples throughout the REP (Figure 2B) as previously reported^27^, ^28^, ^29^, an effect that could result in overestimation of the knockout. To confirm the efficiency of the knockout, we induced maximal surface PD-1 expression post-REP via a 48-h stimulation with aCD3/aCD28 beads, observing a median reduction of 87.53% (min–max: 76.04–97.01%) in edited samples (Figures 2A, 2C, and S2B).

As a further validation, we analyzed the on-target effect of this genetic intervention at the genomic level using the Indel Detection by Amplicon Analysis (IDAA) assay.^30^ This rapid non-sequencing-based method amplifies the genomic area of interest and measures fragment length with single base-discrimination resolution to detect the presence of insertions and deletions (indels) induced by CRISPR-Cas9 treatment. Here, we observed substantial indel formation in the targeted area (median 87.3%) of edited samples at day 14, an effect not seen in mock controls (Figure 2D). Additionally, a strong positive correlation (R = 0.85, R^2^ = 0.72) between indel percentage on REP day 14 and surface PD-1 reduction after post-REP-stimulation was identified (Figure 2E).

### PD-1 CRISPR-Cas9 generates TIL-based ACT products with consistent on- and off-target genomic profiles

High replicability and modification stability are crucial for the advancement of clinical trials involving cellular and gene therapy products.^31^, ^32^, ^33^ Therefore, inducing the same indel pattern mediating the same effects across multiple samples is critical. We conducted a deeper analysis of the indel profile generated by our chosen PD-1-targeting guide RNA (gRNA) in the REPs described above in order to confirm this consistency.

**Figure 3:**
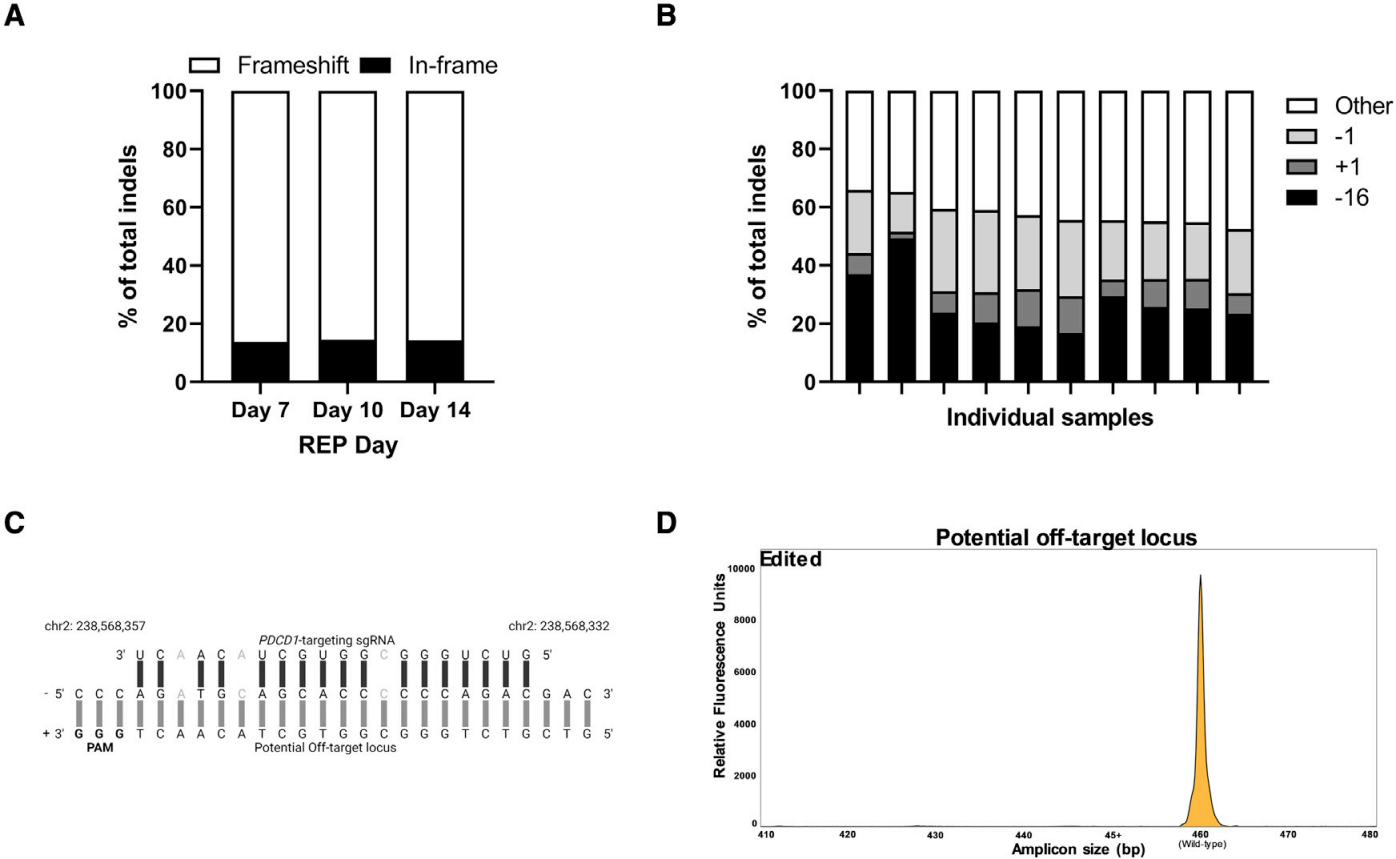
PD-1-targeting CRISPR-Cas9 generates consistent on- and off-target genomic profiles (A) Fraction of frameshift (white bars) and in-frame indels (black) as a percentage of total indels in edited samples throughout the REP. The median of 10 samples for each category is displayed, and each sample is represented by two replicates. (B) REP day 14 contribution of top three indels (−16 bp, black; +1 bp, dark gray; -1 bp, light gray) to total indels shown as percentage of total indels for each sample. Remaining indels are represented by “Other” (white). Each sample is represented by two replicates, and average expression is shown. (C) Base-pair-level depiction of *PDCD1*-targeting gRNA binding to a potential off-target site (black bars). Base-pair mismatches are faded out. Protospacer adjacent motif (PAM) for Cas9 binding is in boldface. Chromosomal location, strand direction, and sense are annotated. Created with BioRender.com. (D) Representative off-target IDAA assay plot from REP day 14 samples. Wild-type amplicon is shown in yellow.

The percentage of in-frame and frameshifting indels, the latter of which is key for an efficient knockout,^34^^,^^35^ was consistent for all edited samples throughout the REP, with frameshifting indels forming median 86.24%, 85.44%, and 85.67% of total indels at days 7, 10, and 14, respectively (Figure 3A). Analysis of the specific indel profile of day 14 edited samples revealed that the majority of indels (median 57.8%) were represented by three specific indels in all samples: +1, −1, and −16 (Figure 3B).

Unwanted off-target editing by CRISPR-Cas9 systems is a major impediment to the implementation of these methods in a clinical setting. As described above, we utilized CRISPR-RNPs delivered by electroporation, a transient setup known to result in few to no off-target effects due to rapid RNP clearance and a non-viral/plasmid transfection method.^36^, ^37^, ^38^ To detect potential activity in off-target loci, we first determined all potential guide target sites with up to four base mismatches. We identified only one off-target site with less than four mismatches (Figure 3C), and IDAA analysis of this site revealed no off-target effects in any edited samples (Figure 3D). Off-target sites with four or more mismatches were not tested due to the greatly diminished possibility of activity.^39^

### PD-1-targeting CRISPR-Cas9 has minimal effects on expanded TIL functionality and phenotype

Having confirmed the on-target efficiency of our knockout and its lack of negative effect on TIL proliferation in the REP, we next explored whether the functionality and phenotype of PD-1 deficient expanded TILs were affected by the knockout.

**Figure 4:**
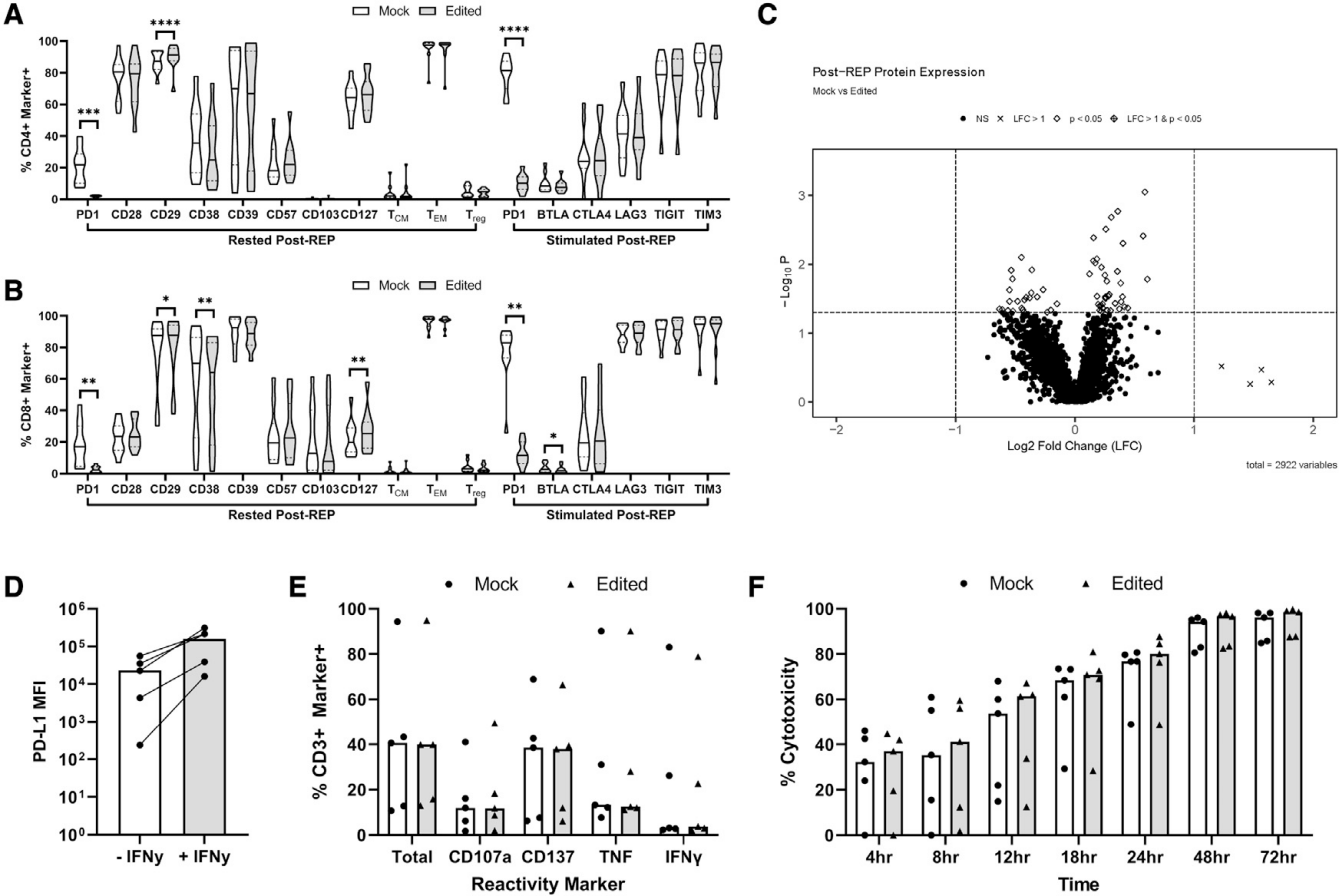
PD-1-targeting CRISPR-Cas9 has minimal effects on expanded TIL functionality and phenotype (A) Flow cytometry phenotype analysis of mock (white) and edited (gray) CD4+ REP-TILs post-REP. Samples were rested and stained or stimulated for 48 h with aCD3/aCD28 beads prior to staining. Markers analyzed can be found on the x axis. T_EM_, effector memory phenotype; T_CM_, central memory phenotype; T_reg_, regulatory phenotype. Solid and dotted lines represent median and quartiles, respectively. The average of two replicates was used for each of the 10 samples. Statistical significance was tested via paired t tests or Wilcoxon matched-pairs signed rank test ((∗p ≤ 0.05, ∗∗p < 0.01, ∗∗∗p < 0.001, ∗∗∗∗p < 0.0001). (B) Same as A but for CD8+ REP-TILs. (C) Volcano plot comparing expression of 2,922 proteins from post-REP mock and edited REP-TILs. Each dot represents average expression (10 samples per condition, 2 replicates per sample) of one identified protein. Crosses signify proteins upregulated (log2 fold change >1) in mock (positive values) or edited (negative values), and diamonds denote statistically significant effects (paired t test, p ≤ 0.05). Differentially expressed proteins match both criteria and are shown as cross within diamond. (D) PD-L1 MFI of autologous tumor cell lines used for reactivity and cytotoxicity experiments, with and without IFNγ treatment. All reactivity and cytotoxicity tests described were carried out with IFNγ-treated cells. (E) Upregulation of reactivity markers in mock and edited CD3+ TILs measured via flow cytometry after 8-h co-culture with PD-L1 expressing autologous tumor cell lines. An average of three replicates was used for each of the five samples. Bars signify median. (F) Cytotoxicity of mock and edited cells during 72-h co-culture with PD-L1 expressing autologous tumor cell lines. Cytotoxicity calculated relative to tumor alone (negative) and cytotoxic agent (positive) controls. An average of three replicates was used for each of the five samples. Bars signify median. (A, B, E, and F) White bars and shaded bars represent mock and edited samples, respectively. (D–F) Experiments were carried out using three MM, one ovarian, and one sarcoma sample (see Materials and methods for details).

Flow analysis of contextually relevant cell surface and intracellular markers revealed few differences between edited and mock control samples post-REP (CD4+/CD8+ subgroups; Figures 4A and 4B). Increased CD29 (p = 0.0371) and CD127 (p = 0.0049), both associated with stemness and persistence,^40^^,^^41^ were observed in edited CD8+ TILs. CD38 expression, a T cell activation marker with conflicting roles in tumor immunology,^42^ was diminished in edited CD4+ and CD8+ TILs (p ≤ 0.0001 and p ≤ 0.0098, respectively). Expression of CD39 and CD103, markers of potentially superior ACT products,^29^^,^^40^ was unaffected by editing. Statistically significant reduced expression of B and T lymphocyte attenuator (BTLA) in edited CD8+ TILs (p = 0.0028) after stimulation with aCD3/aCD28 beads post-REP was detected, a trend also seen in edited CD4+ TILs albeit not to statistical significance. Expression of multiple immune checkpoint molecules (CTLA4, LAG3, TIGIT, TIM3) was unaffected after stimulation. As expected, PD-1 expression was statistically significantly decreased in edited samples at rest and post-stimulation in both subsets.

We then utilized a liquid chromatography-mass spectrometry-based approach (LC-MS, see Materials and methods) to compare the proteome of edited and mock control samples at a larger scale. Of the 2,922 proteins quantified, none were determined to be differentially expressed in either condition (Figure 4C).

Potential effects of PD-1 CRISPR-Cas9 on expanded TIL reactivity and cytotoxicity were tested via co-culture, with autologous tumor cell lines induced to express PD-L1 (Figure 4D). Upregulation of reactivity markers (CD137, IFNƴ, TNF, CD107a) was tested via flow cytometry and was fully maintained in edited cells (Figure 4E). Similarly, cytotoxicity measured over a 72-h co-culture period confirmed the preserved potency of edited cells (Figure 4F).

### PD-1 CRISPR-Cas9 is feasibly and easily integrated into clinical-scale ACT-TIL production

To confirm our pre-clinical-scale model results and validate their application at a clinically relevant scale, we produced a PD-1-deficient GMP-quality clinical-scale infusion product using pre-REP TILs from a patient with head-and-neck cancer. Implementation of PD-1 CRISPR-Cas9 required minimal modification of the existing GMP facility workflow, extending the REP setup process by no more than 1 h.

**Figure 5:**
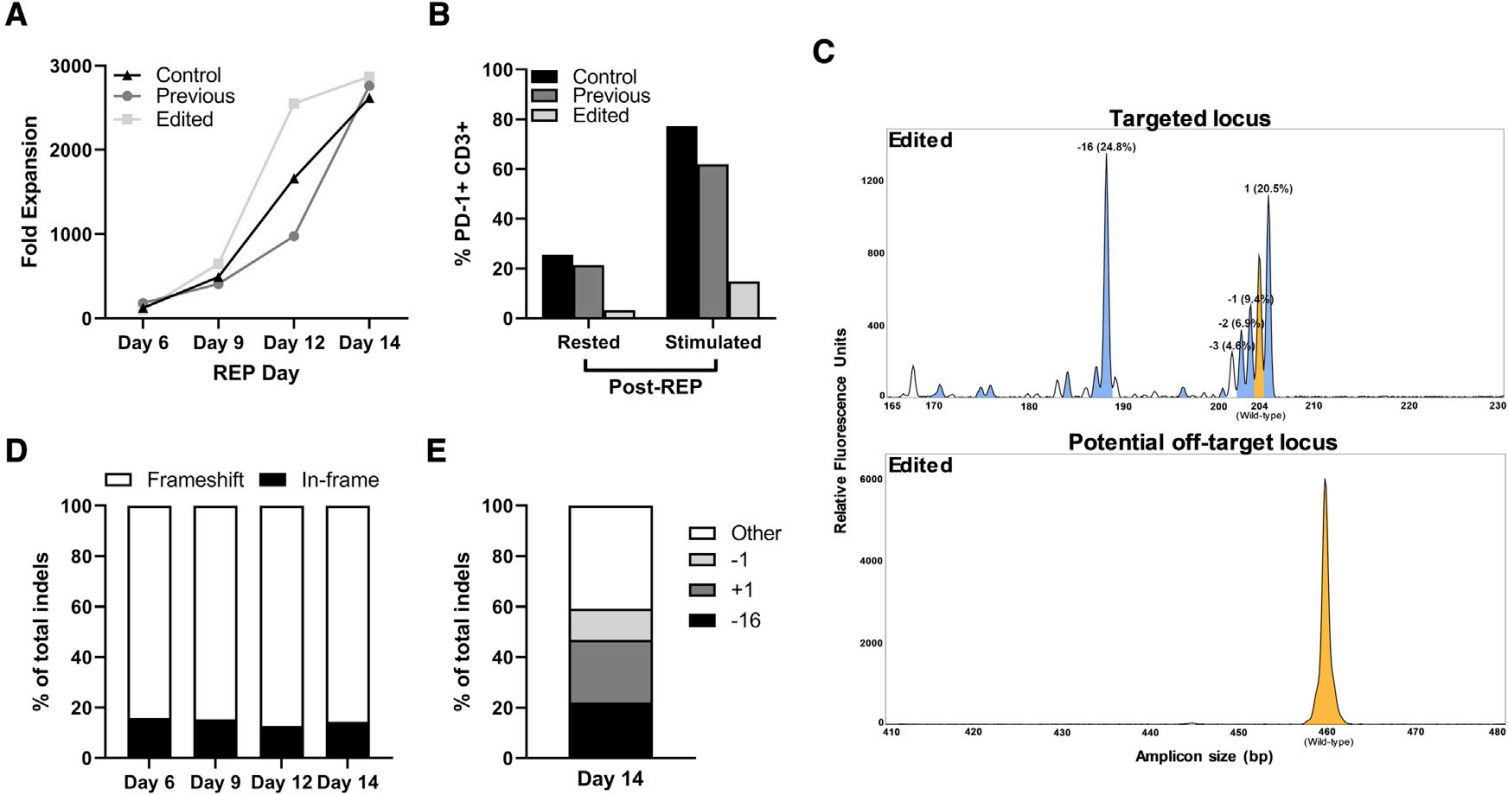
Successful integration of PD-1-targeting CRISPR-Cas9 into a clinical-scale TIL-based ACT workflow (A) Fold expansions throughout a PD-1 CRISPR-Cas9-treated (edited) clinical-scale REP compared with a previous unedited clinical-scale REP and control laboratory-scale samples. Edited sample is shown in light gray squares, previous sample in dark gray circles, and the control in black triangles. (B) Percentage of PD-1+ CD3+ cells measured post-REP via flow cytometry in samples that were either directly stained or stimulated for 48 h with aCD3/aCD28 beads. Coloring as in (A). (C) REP day 14 IDAA on-target (top) or off-target (bottom) plots for the edited sample. Yellow peaks denote unedited wild-type amplicons, blue peaks indicate frameshift indels, and white peaks indicate in-frame indels. (D) Fraction of frameshift (white bars) and in-frame indels (black) as a percentage of total indthree indels (−16 bp, black; +1 bp, dark gray; −1 bp, light gray) to total indels is shown as percentage of total indels. Remaining indels are represented by “Other” (white).

The edited product expanded to clinically administrable levels and corresponded to the control REP and TIL-based ACT product previously generated for patient treatment (Figure 5A), confirming our observations above. Similarly, PD-1 surface protein levels in the edited product were considerably reduced (87.37% rested and 80.92% stimulated; Figure 5B). IDAA analysis of the edited product confirmed the extent of on-target (83.4% indels) and an absence of off-target editing (Figure 5C). The edited sample indel profile was dominated by frameshift indels and remained consistent throughout the REP as expected (Figure 5D), and 59.23% of total indels were represented by the −1, +1, and −16 indels as observed above (Figure 5E).

## Discussion

In this study, we report the use of a rapid, easy-to-use, safe, reproducible, and highly efficient non-viral CRISPR-Cas9 system to generate genetically modified REP-TILs for TIL-based ACT. Our observed editing efficacy at the *PDCD1* locus surpassed that previously reported in this context and was achieved with easily obtainable reagents and minimal workflow modifications.^23^^,^^24^ We anticipate that the combination of highly efficient and user-friendly methods reported here will stimulate further progress in this area and promote the development of the next generation of TIL-based ACT. Specifically, the compatibility of this method with non-MM cancers is of great interest. The reduced response rates of many non-MM solid cancers to immunotherapies such as TIL-based ACT make this an intriguing space for potential gene-editing-mediated enhancements.^8^^,^^10^^,^^43^

The use of CRISPR-Cas9 over other commonly used methods of gene editing such as ZFNs and TALENs provides several benefits. Due to the minimal components required and its RNA-DNA interaction-mediated target recognition mechanics, CRISPR-Cas9 guide design is easier and less time-consuming than its counterparts.^44^ Consequently, many freely available and easy-to-use tools have emerged to facilitate rapid design and *in silico* analysis of potential guides for all user levels.^45^ Following design, the synthesis of CRISPR-Cas9 reagents requires only routine oligo synthesis and cloning procedures—a contrast to the costly and time-consuming complex cloning methods and protein engineering required for TALENs and ZFNs.^44^ This has led to the extensive commercial availability of inexpensive, high-quality CRISPR-Cas9 reagents mediating superior editing efficiencies such as those reported here. Although not investigated here, the application of CRISPR-Cas systems to multiplex editing is less challenging than its rivals.^46^ Multiplex editing is the next logical step in the application of genetic editing to therapeutic settings and is already being tested in ACT contexts.^47^, ^48^, ^49^

Off-target editing is one of the greatest barriers to the broad implementation of CRISPR-Cas-based methods in the clinic, as downstream effects can have serious consequences.^50^ Traditionalefforts have focused solely on detecting targeting of unintended genomic loci, whereas recent studies have shown that unexpected off-target CRISPR-Cas9 editing outcomes can include large deletions and insertions, translocations, inversions, and chromosomal rearrangements.^49^, ^50^, ^51^, ^52^, ^53^ We minimized the risk of these effects by using a rigorous gRNA design process, resulting in only one predicted off-target site (intergenic non-coding DNA) of potential concern (3 mismatches). Importantly, the bulk of off-target reports have utilized viral or plasmid-based systems that can increase off-target events via mechanisms such as continuous Cas9 protein expression^37^^,^^54^ or viral integration.^36^^,^^55^ Our use of a highly transiently expressed, via-electroporation-delivered RNP complexes, engineered Cas9 variant with decreased off-target editing activity (HiFi Cas9)^56^ minimized this risk. RNP complexes have been demonstrated to be almost completely degraded within 24 h of cell entry,^37^ ensuring only a short burst of cellular exposure to Cas9, and electroporation delivers the specified cargo without additional reagents or vectors. We observed no off-targeting editing at the tested site, suggesting that this approach is a reliable choice for use in TIL-based ACT. On-target editing profiles must also be considered for clinical applications, as erratic indel formation may lead to inconsistent knockouts or unforeseen effects. It has been observed that on-target indel profiles are often gRNA specific and consistently reproducible across samples,^57^ and we confirmed this stability in our samples. It has more recently been suggested that the predictability of Cas9-induced double-stranded break repair depends on the target site,^58^ indicating that although our chosen target site is clearly repaired in a consistent stable manner, alternative target sites may not behave correspondingly and must be carefully validated.

The potential negative effects of gene editing are key factors that may restrict the application of genetic editing to TIL-based ACT. For example, it is well known that both genomic damage and electroporation can induce cell death.^59^^,^^60^ Despite indications of these effects, our observed recovery rate was sufficient for standard manufacturing of clinically relevant numbers of TILs ready for infusion. Total fold expansions were unaffected by gene editing across histologies, an important finding for potential pan-histology applications given that the number of cells infused into a patient has been reported to correlate with response to therapy.^61^^,^^62^ These studies have demonstrated CD8+ TILs to be the driver of this correlation, and we observed a minor shift in the CD4/CD8 ratio partially mediated by a slight reduction of the CD8+ fraction in edited samples. However, this effect was not observed in all samples and may simply be an artifact induced by small sample size or a target-specific effect of PD-1 knockout.

The effects of this knockout on cellular phenotype are currently unclear. Previous studies have observed PD-1 inhibition to result in a compensatory upregulation of additional immune checkpoints^63^^,^^64^; however, we did not observe this phenomenon after our PD-1 knockout. Recently, Lu et al. reported the unique transcriptomic profile of TIL-based ACT-infused CD8+ REP-TILs persisting for more than 40 days in a patient post-infusion.^41^ Similarly, Krishna et al. reported the importance of CD8+CD39–CD69– cells for responses to TIL-based ACT.^40^ Both reports identified increased CD29 and CD127 expression in the key population, a pattern also observed in our total PD-1 knockout samples. We also detected reduced expression of CD38 in PD-1 knockout samples, as seen in the stem-like self-renewing CD8+CD39–CD69– population by Krishna et al.^40^ These observations suggest that PD-1 knockout may improve the quality of the product; however, it should be noted that these subsets were not characterized in detail with this methodology and that the effect sizes we report are small. Moreover, LC-MS-based proteomic comparison did not confirm the changes observed, although only a fraction of the total proteome was quantifiable, and this methodology did not differentiate surface and total protein.

Studies have reported the increased IFNγ secretion and cytotoxic capabilities of PD-1-deficient cells following *in vitro* stimulation.^23^^,^^47^^,^^65^^,^^66^ We were unable to replicate these findings, although this may have been due to the system employed. T cell co-stimulation via CD28-CD80/CD86 interactions is required to observe the effects of anti-PD-1 therapies,^67^^,^^68^ and we observed little to no expression of these molecules on the tumor cells used (data not shown). Consequently, it is feasible that these benefits could be observed if an alternative model were employed. The increased CD29 expression that we observed in the CD8+ compartment has also been linked with increased cytolytic and IFNƴ-secreting ability.^69^

PD-1 is a logical target for this application, yet reports are divided on the exact benefits of PD-1 inhibition on T cells.^23^^,^^47^^,^^70^^,^^71^ Indeed, there are multiple factors that may preclude its effectiveness in TIL-based ACT. The observed loss of PD-1 expression throughout the REP could severely reduce the effect sizes of any treatment targeting this axis in a TIL-based ACT context and may have contributed to the minimal functional effects we report here. Yu et al. recently demonstrated that TCR and PD-1 signaling promoted the accumulation of damaged mitochondria in CD8+ TILs, resulting in increased exhaustion and impeded functionality.^72^ The same study also reported a higher percentage of damaged mitochondria in TILs from advanced tumor stages. As TIL-based ACT is primarily employed as a second-line or later therapy due to a lack of regulatory approval, advanced tumor stages and long disease courses are common.^3^^,^^4^ It is therefore conceivable that considerable damaging PD-1 signaling has already occurred prior to the CRISPR-Cas9 intervention that we report here. Finally, recent reports have indicated that the effects of checkpoint inhibitor therapy (CPI) therapy may be mediated by an influx of “fresh” T cells rather than exhausted TILs already present in the tumor microenvironment,^73^^,^^74^ thereby potentially rendering the described intervention superfluous.

The increased utilization of genetic editing will be crucial for the development of the next generation of TIL-based ACT, and we believe our demonstration of highly efficient, easy-to-use CRISPR-Cas9 demonstrates the suitability of this methodology for TIL-based ACT. It should serve as both a foundation and a stimulus for similar efforts in the future.

## Materials and methods

### Cellular material

Fresh tumor material was obtained via biopsy or surgical resection for enrollment in clinical trials (ethical approval reference: H-20070020 or previously reported) or standard treatment at the National Center for Cancer Immune Therapy (CCIT-DK), Department of Oncology, Copenhagen University Hospital, Herlev, Denmark.^29^^,^^75^, ^76^, ^77^, ^78^, ^79^ Pre-REP TILs were isolated and expanded *in vitro* from tumor fragments by using a two-step process previously described.^43^^,^^77^, ^78^, ^79^, ^80^ Rapid-expansion protocol TILs (REP-TILs) were generated from frozen pre-REP TILs via the REP (described below). Autologous *in vitro* cultured tumor cell lines (TCLs) were established via serial passage of adherent cells from tumor fragments deriving from the same lesions from which TILs were cultured.^81^ TCLs were authenticated via *in vitro* growth patterns, morphology (light microscopy), and lineage antigen expression (PCR) where necessary. Mycoplasma testing (cat. no. A3744.0020; VWR International, Lutterworth, UK) was carried out according to the manufacturer’s protocol and was negative. All procedures were performed in compliance with the clinical protocols approved by the Ethics Committee of the Capital Region of Denmark and national regulations for biomedical research. Unless stated otherwise, all experiments were carried out using five MM and five non-MM (MSS-colorectal, MSI-colorectal, ovarian, head-and-neck, and thyroid cancer) samples.

### CRISPR-Cas9 RNP complex formation and delivery

The *PDCD1* targeting guide (5′-GTCTGGGCGGTGCTACAACT-3′) was designed using the CRISPick,^82^^,^^83^ E-CRISP,^84^ CHOPCHOP,^85^ and Custom Alt-R CRISPR-Cas9 guide RNA (Integrated DNA Technologies [IDT], Coralville, IA) design tools. Our optimized setup utilized S.p. HiFi Cas9 Nuclease V3, single guide RNAs, and electroporation enhancer (EE) from IDT’s Alt-R CRISPR-Cas9 catalog. Negative control crRNA (crispr-RNA) #2 from the same catalog was used for “Mock” controls. Nuclease-Free Duplex Buffer (IDT) was used to resuspend and dilute all reagents. Mock single guide RNAs were formed by mixing the crRNA with trans-activating crispr-RNA (IDT) at an equimolar ratio for 5 min at 95°C and then cooling to room temperature. RNPs were formed fresh prior to the reaction by mixing single guide RNAs with Cas9 nuclease at a 2.5:1 ratio (8 μL total volume) for 15 min at room temperature. two microliters of EE was then added and rested for 1 min and 90 μL of cells was added to the RNP/EE mixture and again rested for 1 min before transferring the entire 100-μL volume to a 2-mm electroporation cuvette (Harvard Apparatus, Holliston, MA) and immediately electroporating with the ECM 830 Square Wave Electroporation System (250 V, 2 ms, 1 pulse; BTX Molecular Delivery Systems, Holliston, MA). Cells were then gently and rapidly transferred to pre-warmed complete medium (CM, RPMI- 1640 plus GlutaMAX and 25 mM HEPES (Gibco, Thermo Fisher Scientific, Waltham, MA), 10% heat-inactivated human AB serum (Sigma-Aldrich/Merck KGaA, Darmstadt, Germany), 6,000 IU/mL rhIL-2 (Proleukin, Novartis, Basel, Switzerland), Fungizone (Bristol-Myers Squibb, New York, NY), 100 U/mL penicillin, and 100 μg/mL streptomycin (Gibco)). Each 100-μL reaction contained 2 × 10^6^ – 25 × 10^6^ cells, 2.5 μM RNPs, and 5 μM EE.

### Rapid expansion protocol

REP-TILs were thawed and rested in CM for 48 h prior to the REP. On day 0 of the REP, cells were harvested, washed twice with Opti-MEM I Reduced Serum Medium (Gibco), and electroporated with CRISPR or Mock RNPs, as described above. Cells were then rested for 1 h prior to starting the 14-day REP protocol. Briefly, pre-REP-TILs were cultured with 6,000 IU/mL rhIL-2, 30 ng/mL anti-CD3 antibody (Orthoclone OKT3, Cilag AG, Schaffausen, Switzerland; or MACS GMP CD3 pure, Miltenyi Biotec, Bergisch Gladbach, Germany) and irradiated allogenic peripheral blood mononuclear feeder cells from 12 donors (1:200 TIL:Feeder). Pre-REP-TILs (0.1 × 10^6^ or 20 × 10^6^) were used to begin the pre-clinical-scale and clinical-scale REPs, respectively. Cell counts were carried out using a hemocytometer (Hausser Scientific, Horsham, PA) and 0.1% trypan blue (Sigma-Aldrich/Merck KGaA) for dead cell exclusion. The full REP protocol has previously been described in detail.^26^^,^^81^

### Phenotyping

All flow cytometry samples were acquired on a NovoCyte Quanteon (ACEA Biosciences, San Diego, CA) or FACSCanto II (BD Biosciences, Franklin Lakes, NJ). See supplemental methods for detailed description of flow cytometry antibodies utilized. An example of PD-1 expression gating strategy can be found as Figure S3. All antibody staining-related incubations took place in the dark at 4°C.

Surface PD-1 expression during the REP was measured by taking samples directly from the REP at specified time points. Cells were washed with Dulbecco’s phosphate-buffered saline (Sigma-Aldrich/Merck KGaA), stained with the antibody cocktail for 25 min, washed again, and acquired.

For phenotyping of unstimulated REP-TILs post-REP, cells were thawed and rested for 24 h in TIL medium (RPMI 1640 plus GlutaMAX and 25 mM HEPES, 10% heat-inactivated human AB serum, 100 U/mL penicillin, 100 μg/mL streptomycin). Cells were then harvested and stained for extracellular and in some cases intracellular markers. Panels detecting only extracellular markers were stained and acquired as described above. Panels additionally detecting intracellular markers were processed for extracellular markers as normal and then subsequently processed for intracellular staining using the eBioscience Intracellular Fixation & Permeabilization Buffer Set (Invitrogen, Thermo Fisher Scientific, Waltham, MA). Briefly, after the final extracellular wash, cells were incubated with fixation buffer for 30 min, before being washed twice with permeabilization buffer, and then stained with an intracellular marker-targeting antibody cocktail for 30 min. Cells were then washed once more with permeabilization buffer and acquired as above.

For phenotyping of stimulated REP-TILs post-REP, cells were thawed and rested as above before being stimulated with Dynabeads Human T-Activator CD3/CD28 for T cell expansion and activation (Gibco). Cells were seeded 1.5 × 10^6^ per well in a 48-well plate with Dynabeads at a 1:1 ratio and 500 IU/mL rhIL-2 for 48 h (37°C, 5% CO_2_). Cells were then harvested, stained for extracellular and intracellular markers, and acquired as above.

PD-L1 expression on autologous TCLs was determined after stimulation with or without 100IU/ml IFNƴ. Cells were harvested after 72 h of stimulation and then stained as above. An isotype control was used to control for non-specific background staining by subtracting the isotype median fluorescence intensity values (MFI) from the test MFI.

### Anti-tumor reactivity

The reactivity of PD-1 knockout REP-TILs against patient-derived *in vitro* cultured autologous TCLs was tested by measuring expression of IFNƴ, TNF, CD107a, and CD137 via flow cytometry after co-culture. TCLs were pre-treated for 72 h with 100IU/ml IFNγ. TILs and TCLs were co-cultured for 8 h at a 3:1 ratio in a round-bottom 96-well plate in the presence of anti-CD107a staining antibody, brefeldin A (BD GolgiPlug, 1:1,000), and Monensin (BD GolgiStop, 1:1,000) (all BD Biosciences). The mixture was then harvested and stained via intracellular staining as described above in Phenotyping. The specific flow cytometry antibodies utilized can be found in the supplemental methods. Cytotoxicity experiments were carried out with REP-TILs from protocol optimization experiments (3 MM, 1 ovarian, 1 sarcoma, ∼70% knockout; data not shown) and were not repeated with optimized knockout REP-TILs due to the lack of effect observed and lack of matching autologous tumor cell lines. Reactivity was calculated using TILs cultured without stimulation as a control.

### Tumor cytotoxicity

Cytotoxicity of PD-1 knockout REP-TILs was tested using the impedance-based xCELLigence Real-Time Cell Analysis SP instrument (ACEA Biosciences, San Diego, CA) according to the manufacturer’s instructions.^86^ Briefly, patient-derived *in vitro* cultured autologous TCLs pre-treated with 100 IU/mL IFNγ (PeproTech, Hamburg, Germany) for 48 h were seeded in wells of an RTCA E-Plate 96 PET plate (ACEA Biosciences) and incubated for 24 h (5% CO_2_, 37°C) to allow attachment to the well surface. REP-TILs were then added, and killing was monitored via changes in well-surface impedance. Cytotoxicity was then calculated relative to positive (100% cytotoxicity, 1% Triton X-100 [Sigma-Aldrich/Merck KGaA]) and negative controls (0% cytotoxicity, tumor alone). Cytotoxicity experiments were carried out with REP-TILs from protocol optimization experiments (3 MM, 1 ovarian, 1 sarcoma, ∼70% knockout; data not shown) and were not repeated with optimized knockout REP-TILs due to the lack of effect observed and lack of matching autologous tumor cell lines.

### Indel detection by amplicon analysis

Cell pellets for indel detection by amplicon analysis (IDAA) were frozen throughout the REP by centrifuging (5 min, 500 × *g*, 4°C), discarding supernatant, and storing immediately at −80°C. Pellets were thawed and processed with CoboXtract Quick DNA Extraction Solution (Cobo Technologies, Copenhagen, Denmark) according to the manufacturer’s instructions. Cell lysate (1μL) was used as template for tri-primer amplification using a universal 5′-FAM-labeled universal FAMFOR primer (5′-6-FAM-AGCTGACCGGCAGCAAAATTG-3′) as described previously.^30^ In brief, PD1 tri-primer amplification was carried out using Profilase polymerase (Ampliqon, Odense, Denmark) and PD1FOR3EXT (5′-AGCTGACCGGCAGCAAAATTGcaccctcccttcaacctgac-3′)/PD1REV3B (5′-ccgaccccacctacctaagaacc-3′)/FAMFOR primers as recommended by the manufacturer. For off-target analysis (chr2:238,568,240-238,568,446), primers PDCDOT1FOREXT4 (5′-AGCTGACCGGCAGCAAAATTGcatcccctctccacctgctagag-3′)/PDCDOT1REV4 (5′-gctcttgattcagcagatgcagggc-3′) (artificial bases shown in bold and FAMFOR primer extension in upper case) and FAMFOR were used. A touch-down thermocycling profile with a final annealing temperature of 58°C was used for both on-target and off-target tri-primer amplification. Tri-primer PCR products were diluted 1:60 and 1:180, mixed with 0.5-μL LIZ600 ladder (Applied Biosystems, Thermo Fisher Scientific, Waltham, MA), and loaded onto an ABI3500XL (Applied Biosystems) for fragment analysis. Raw.fsa data files were analyzed using the ProfileIT software package (https://viking-suite.com/).

### SWATH liquid chromatography-mass spectrometry

REP-TILs frozen at REP day 14 were thawed and rested in TIL medium for 24 h before extraction of total protein using the PIPPR Total Mammalian Protein Extraction Kit (Cobo Technologies) per the manufacturer’s instructions. Extracted protein was then further processed and analyzed using a SWATH liquid chromatography-mass spectrometry approach by Cobo Technologies (see Supplemental methods).

### Statistical analyses and data analysis software

Statistical analyses and graphs were generated using GraphPad Prism v.9.0.0 (GraphPad Software, San Diego, CA) or R Studio (1.4.1717, RStudio, Boston, MA). Negative values deriving from the subtraction of unstimulated samples from stimulated samples were converted to 0.01% for statistical analyses and figure generation. A limit of detection of 0.5% was applied to all flow cytometry data, meaning all values below this threshold were converted to 0.5% for statistical analyses and figure generation. Values exceeding 100% after normalization due to previous background subtraction were converted to 100% for statistical analyses and figure generation. For tests of statistical significance, normality was tested and the appropriate statistical test then applied. The specific statistical test or regression employed is specified in the relevant figure legend. Only statistically significant values are marked on figures. All values are expressed as median unless otherwise specified. NovoExpress v.1.4 (ACEA Biosciences) and FlowJo v.10.6 (FlowJo LLC, Ashland, OR) were used to analyze flow cytometry data.

### Data availability

Data supporting the results and conclusions presented are available upon reasonable request. Requests should be directed to the corresponding authors, and access will be provided according to the institutions applicable policies and laws. As this study involves patient samples, sharing of data may require agreements such as Data Processing Agreements prior to data sharing.

## Supplemental information

Document S1. Supplemental methods and Figures S1–S3

Document S2. Article plus supplemental information
