# Supplementary material for: Highly efficient PD-1-targeted CRISPR-Cas9 for tumor-infiltrating lymphocyte-based adoptive T cell therapy
Source: Mol Ther Oncolytics. 2022 Jan 10;24:417–28. doi: 10.1016/j.omto.2022.01.004 (PMC8807971; doi:10.1016/j.omto.2022.01.004)
Supplement: Document S1. Supplemental methods and Figures S1–S3 [file mmc1.pdf]

OMTO, Volume *24*

Supplemental information

Highly efficient PD-1-targeted CRISPR-Cas9 for tumor-infiltrating lymphocyte-based adoptive T cell therapy

Christopher Aled Chamberlain, Eric Paul Bennett, Anders Handrup Kverneland, Inge Marie Svane, Marco Donia, and Özcan Met

Supplemental Methods

List of flow cytometry antibodies used as described in Methods (sections Phenotyping, Anti-tumor reactivity).

Marker	Colour	Vendor	Cat #
BTLA	PE	BioLegend	344506
CCR7	PE	BioLegend	353204
CD103	FITC	BD Biosciences	550259
CD107a	BV421	BD Biosciences	562623
CD127	VioBright FITC	Miltenyi Biotec Inc.	130-113-417
CD137	PE	BD Biosciences	555956
CD25	BV421	BD Biosciences	564033
CD28	APC	BD Biosciences	559770
CD29	PE	BD Biosciences	555443
CD3	BV510	BD Biosciences	740202
CD3	PE-CF594	BD Biosciences	562280
CD3	BV786	BD Biosciences	563800
CD38	PE-Cy5	BD Biosciences	555461
CD39	PE-Cy7	Invitrogen	25-0399-42
CD4	BV711	BD Biosciences	563028
CD4	BV510	BD Biosciences	562970
CD45RA	BV650	BD Biosciences	563963
CD57	BV421	BD Biosciences	563896
CD8	Qdot 605	Invitrogen	Q10009
CD8	BV421	BD Biosciences	562425
CD8	APC-R700	BD Biosciences	565165
CTLA4	APC	BioLegend	369612
FoxP3	PE-CF594	BD Biosciences	562421
IFNγ	BV510	BioLegend	502544
LAG3	FITC	BioLegend	369308
LiveDead	Near-InfraRed	Invitrogen	L34976A
PD-1	PE-Cy7	BioLegend	329918
PD-1	PE-CF594	BioLegend	329940
PD-L1	PE-Cy7	BD Biosciences	329918
TIGIT	BV421	BD Biosciences	747844
TIM3	BV711	BD Biosciences	565566
TNF	APC	BD Biosciences	554514

Antibodies were purchased from BD Biosciences (Franklin Lakes, NJ), BioLegend, Miltenyi Biotec Inc. (Bergisch Gladbach, Germany) or Invitrogen (Thermo Fisher Scientific, Waltham, MA) as specified.

SWATH liquid-chromatography mass-spectrometry (LC-MS)

REP-TILs frozen at REP day 14 were thawed and rested in TIL media for 24hr, before extracting total protein using PIPPR Total Mammalian Protein Extraction Kit (Cobo Technologies, Copenhagen, Denmark) as per the manufacturer's instructions. Extracted protein samples were then centrifuged at 18,000 x g, 4°C, for 10 min. The pellet was resuspended in 0.2% RapiGest SF (Waters, Milford, MA) and the total protein content was measured for each sample using the Qubit protein assay kit (Invitrogen, Thermo Fisher Scientific, Waltham, MA). For each sample, 40μg of protein was digested following the RapiGest[1] protocol. Briefly, samples were incubated with DTT (30 min at 60°C) and Iodoacetamide (30 min, room temperature and dark), and then incubated (37°C) with trypsin (Promega, Madison, WI) at 1:40 trypsin to protein ratio in two steps (2 hours plus 15 hours). After digestion, peptide samples were diluted with 0.1% formic acid (FA) in water to 100 ng/μL and transferred to a low-volume HPLC vial.

Peptide samples were then blended into three distinct pools: an equal mix of all CRISPR samples, an equal mix of all mock samples, and an equal mix of all samples. These 3 pooled samples were analysed by LC-MS using a hybrid quadrupole-TOF mass spectrometer (Tims-TOF Pro, Bruker, Billerica, MA) connected on-line to nano-HPLC (nanoElute, Bruker). 200 ng were injected for each pooled sample. The chromatographic separation was performed on a Bruker Fifteen column (150 x 0.075 mm, 1.9μm particle size) at 40°C. The flow rate was set to 300nL/min, and the gradient consisted on 2 – 25% of B (0 – 50 min) and 25 – 35% of B (50-60 min), with mobile phase A being 0.1% FA in water and B 0.1% FA in acetonitrile. As the peptides eluted from the chromatography, they were analysed in the mass spectrometer with a data-dependent acquisition parallel accumulation serial fragmentation (DDA-PASEF) method, where peptides are isolated and fragmented according to their mass/charge (m/z) and ionic mobility values. The interface between the LC and MS was a Captive nano-electrospray source (Bruker) operating at 1500 V and 180°C. The DDA-PASEF method consisted of 10 MS/MS PASEF scans per topN acquisition cycle, with an accumulation time of

100ms and a ramp of 100ms. MS and MS/MS spectra were acquired in an m/z range from 100 to 1700 and in an ion mobility range (1/K0) from 0.60 to 1.60 V s/cm2, selecting precursor ions for the MS/MS PASEF scans from a previous TIMS-MS scan. The collision energy was programmed as a function of ion mobility, following a straight line from 20 eV for 1/K0 of 0.6 to 59 eV for 1/K0 of 1.6. The TIMS elution voltage was linearly calibrated to obtain 1/K0 values using three ions from the ESI-L Tuning Mix (Agilent, Santa Clara, CA) (m/z 622, 922, 1222).

For obtaining the peptide spectral library, the FragPipe[2] computational platform was used, comprising three sequential steps: (i) the DDA-PASEF files were combined in one protein identification search using the MSFragger (version 3.2) database search engine (database: human reference proteome downloaded from UniProt on February 2021; oxidation of methionine, acetylation of protein N-termini, were set as variable modifications; carbamidomethylation of cysteine was set as a fixed modification); (ii) peptides identified were validated, protein were inferred and filtered (1% false discovery rate, FDR) using Philosopher (version 3.4.13) ; and (iii) the spectral library was generated after nonlinear retention time and ion mobility alignment with EasyPQP (version 0.1.13).

Each individual sample (200 ng injections) was analysed by LC-MS using the same LC-MS platform as for the DDA-PASEF runs, but with a shorter gradient (3 – 25% of B (0 – 40 min) and 25 – 30% of B (40-45 min)) and a diaPASEF[3] method. The diaPASEF runs were processed with DIA-NN[2] (version 1.7.16) using the library created with FragPipe. Protein inference in DIA-NN was disabled to use the protein groups assembled at the spectral library building stage in FragPipe. Match-between-runs (MBR) options was enabled. Peptide length range was set from 6 to 30 residues. Quantification mode was set to "Robust LC (high precision)". All other DIA-NN settings were left default.

The library was constructed based on the DDA-PASEF runs acquired for the three pools of samples, and 2958 proteins were included in the library and thus considered for the relative quantification procedure. After extraction of the quantitative data for the 19 diaPASEF runs, a total of 2923 proteins could be quantified. For finding statistically significant abundance changes between the two groups, fold-changes, and p-values (two-tailed t-test on the log2 transformed quantitative values) were calculated for each quantified protein.

Supplemental References

1. Vowinckel, J., Capuano, F., Campbell, K., Deery, M.J., Lilley, K.S., and Ralser, M. (2013). The beauty of being (label)-free: sample preparation methods for SWATH-MS and next-generation targeted proteomics. F1000Research *2*, 272.

2. Demichev, V., Yu, F., Teo, G.C., Szyrwiel, L., Rosenberger, G.A., Decker, J., Kaspar-Schoenefeld, S., Lilley, K.S., Mülleder, M., Nesvizhskii, A.I., et al. (2021). High sensitivity dia-PASEF proteomics with DIA-NN and FragPipe. bioRxiv, 2021.03.08.434385.

3. Meier, F., Brunner, A.D., Frank, M., Ha, A., Bludau, I., Voytik, E., Kaspar-Schoenefeld, S., Lubeck, M., Raether, O., Bache, N., et al. (2020). diaPASEF: parallel accumulation–serial fragmentation combined with data-independent acquisition. Nat. Methods *17*, 1229–1236.

Supplemental Figures

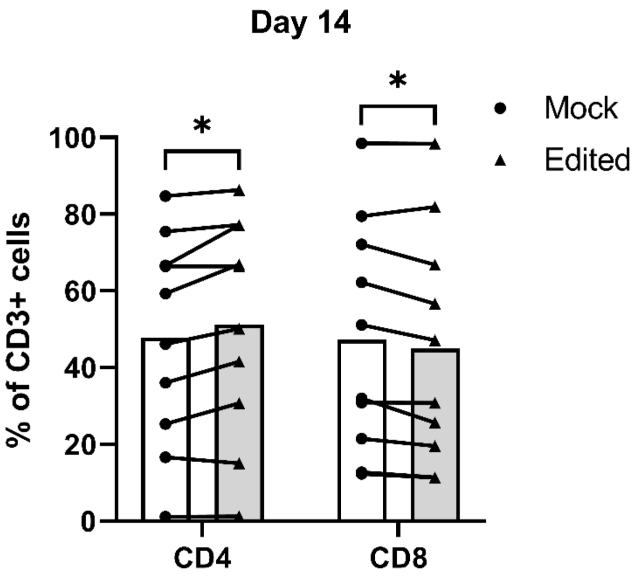

Supplemental Figure 1. Changes in prevalence of CD4+ and CD8+ subsets at REP day 14.
Percentage of CD4+ and CD8+ cells in total CD3+ population in day 14 REP samples. Statistical significance calculated via paired T test ($p<0.05$*). Paired samples are linked with connecting line. Each point represents the average of two replicates per sample. Mock samples are shown as black dots on a white bar and Edited samples as black triangles on a shaded bar. Bars signify median of 10 samples. Corresponds to Figure 1F.

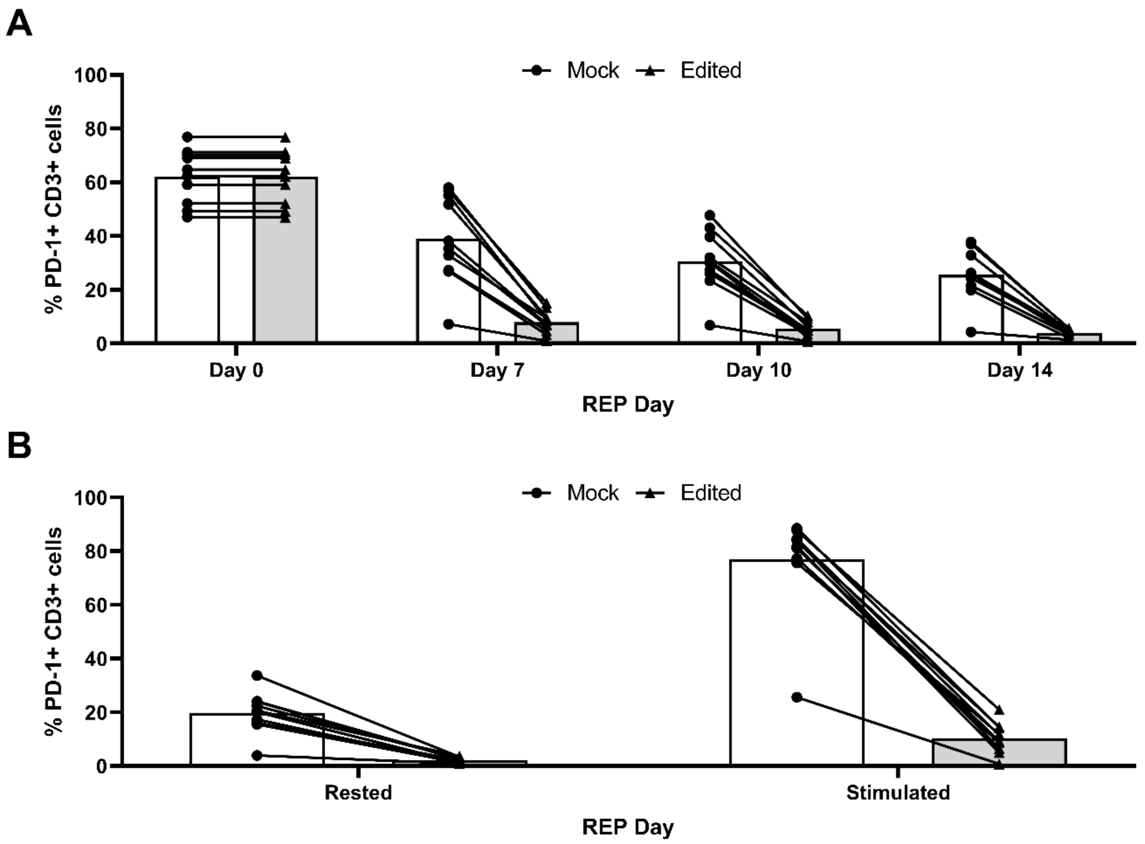

Supplemental Figure 2. PD-1 expression of individual samples pre-, during, and post-REP.
Surface expression of PD-1 on CD3+ TILs from Mock and Edited samples pre-, during, and post-REP, measured via flow cytometry. Paired samples are linked with connecting line. Each point represents the average of two replicates per sample. Mock samples are shown as black dots on a white bar and Edited samples as black triangles on a shaded bar. Bars signify median of 10 samples.

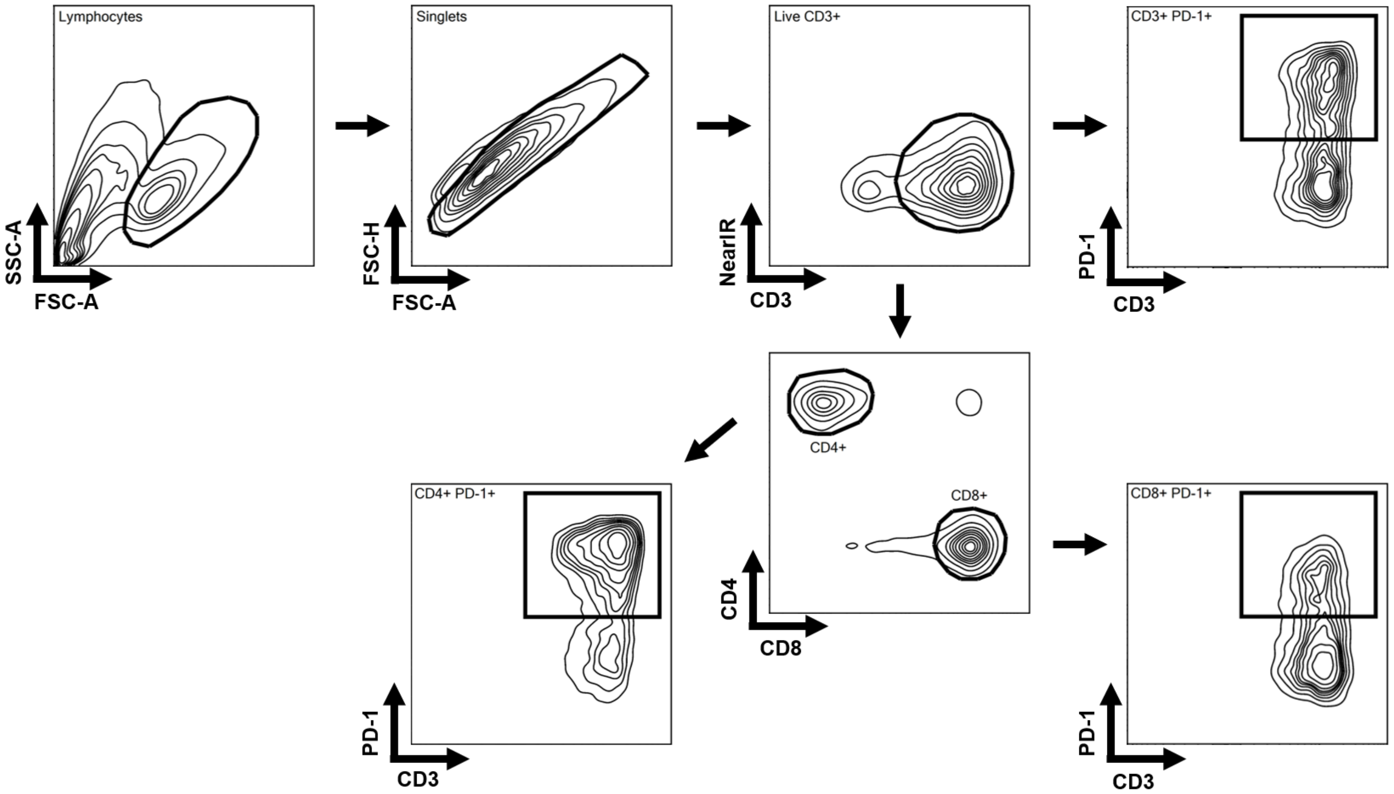

Supplemental Figure 3. Flow cytometry gating strategy for PD-1 detection.
First lymphocytes were selected on a forward scatter area (FSC-A) vs side scatter area (SSC-A) plot. Doublets were then excluded using a FSC-A vs forward scatter height (FSC-H) plot. Live CD3+ cells were then selected to be CD3+ and Near-IR- (viability marker staining dead cells). CD3 cells were then analysed for PD-1 expression (CD3 vs PD-1) or further subgated for CD4 and CD8 expression (CD4 vs CD8). PD-1 expression on CD4/CD8+ cells was gated on CD4/CD8 vs CD4 plots. A list of all antibodies used can be found in the Methods.
